# Research Hotspots and Trends Analysis of Patellar Instability: A Bibliometric Analysis from 2001 to 2021

**DOI:** 10.3389/fsurg.2022.870781

**Published:** 2022-05-16

**Authors:** Zitian Zheng, Wennan Xu, Qingyun Xue

**Affiliations:** ^1^ Department of Orthopedics, Beijing Hospital, National Center of Gerontology, Institute of Geriatric Medicine, Chinese Academy of Medical Sciences, Beijing, China; ^2^ Fifth School of Clinical Medicine, Peking University, Beijing, China

**Keywords:** Bibliometrics, visualized study, patellar instability, patellofemoral joint, patellar dislocation

## Abstract

**Background:**

Patellar instability is a common multifactorial disease in orthopedics, which seriously affects the quality of life. Because of the unified pathogeny, diagnosis and treatment, patellar instability has gradually attracted the interest of more scholars these years, resulting in an explosive growth in the research output. This study aims to summarize the knowledge structure and development trend in the field from the perspective of bibliometrics.

**Methods:**

The data of articles and reviews on patellar instability was extracted from the Web of Science database. The Microsoft Excel, R-bibliometrix, CiteSpace, VOSviewer, Pajek software are comprehensively used to scientifically analyze the data quantitatively and qualitatively.

**Results:**

Totally, 2,155 papers were identified, mainly from North America, Western Europe and East Asia. Until December 31, 2021, the United States has contributed the most articles (1,828) and the highest total citations (17,931). Hospital for Special Surgery and professor Andrew A Amis are the most prolific institutions and the most influential authors respectively. Through the analysis of citations and keywords based on a large number of literatures, “medial patellofemoral ligament construction”, “tibial tubercle-trochlear groove (TT-TG) distance”, “epidemiological prevalence”, “multifactor analysis of etiology, clinical outcome and radiographic landmarks “ were identified to be the most promising research directions.

**Conclusions:**

This is the first bibliometric study to comprehensively summarize the research trend and development of patellar instability. The result of our research provides the updated perspective for scholars to understand the key information in this field, and promote future research to a great extent.

## Introduction

Knee joint is the largest compound joint in human body. Patellofemoral instability as a common disease in orthopedics and the main cause of anterior knee pain syndrome, especially in young people, often leads to patellar dislocation or subluxation, which brings great pain to patients (1). The multiply risk factors leading to patellar instability include the dysplasia of patellar and trochlear osseous structure, the integrity and balance of periarticular ligaments, the systemic conditions affecting connective tissue, and the overall muscle strength, which also complicate the diagnosis and analysis of patellar instability (2). Patellar dislocation leads to pain, decreased mobility, osteochondral fractures, and patellofemoral arthritis (3). The complexity of the pathogenesis also leads to the diversity of treatment methods, and there is no unified opinion on the choice of conservative or surgical treatment, as well as on the choice of surgical procedure (4).

In view of the above aspects, patellofemoral instability has attracted more and more attention of researchers, and a large number of relevant studies have been published. However, the rapidly increasing number of publications makes it increasingly difficult for researchers to keep up with the latest findings, even in their professional field (5). Although the emerging systematic evaluation and meta-analysis can provide researchers with more ideas, these articles only focus on some specific aspect (4, 6). Some meaningful information, such as the number of publications, the collaboration of countries, institutions and authors, and the scientific analysis of research hotspots, prediction of hotspots are not included (7).

Bibliometrics based on scientific statistical methods can reliably identify and analyze the above information, so as to identify global trends and build knowledge structure, which is not only beneficial to novice researchers, but also to the experts (8). Bibliometric analysis has been applied in medical field, exerting a great impact in Orthopedics (9), Psychiatry (10, 11), Urology (12) and other fields.

**Figure 1:**
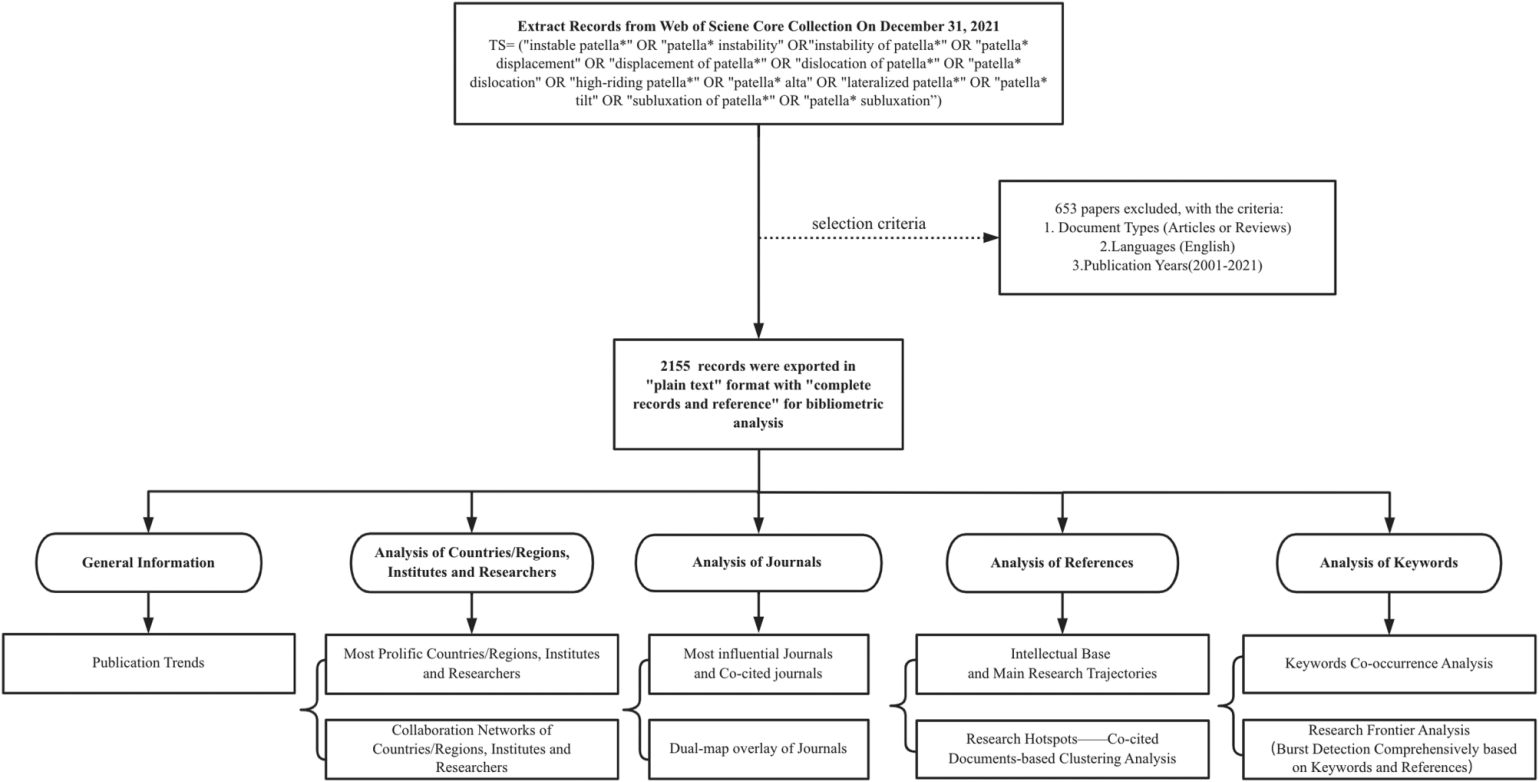
The work flow diagram.

As shown in Figure 1, our analysis mainly contains five aspects. Firstly, we analyze the growth trend of the number of publications. Then we analyze the most productive countries, institutions and researchers, which helps researchers find the most suitable research institutions or scholars to cooperate. Then there is the analysis of journals, including journal co-citation analysis and the dual-map overlay visualization, which helps researchers find the most suitable journals for study and submission. The most prominent part of our research is the analysis of references and keywords. We conduct main path analysis and co-citation cluster analysis on references, which are helpful to clarify the development process and research progress in this field. The co-occurrence analysis of keywords identifies the relationship between various research directions in this field. In addition, we use multiple analysis software to comprehensively analyze keywords to identify the research frontiers and development directions in this field, which may inspire researchers and guide researchers to make more breakthroughs in these promising directions.

## Review on Previous Literature

In order to avoid duplicated and unnecessary investment of research funds and time, the researchers independently and systematically reviewed the Clarivate Analytics Web of Science (WOS) database on the last day of 2021, that is, December 31, 2021. The WOS database is the selected collection of high-quality academic peer-reviewed literatures. We found that in the research field of patellar instability, no bibliometric articles have been published. Bibliometrics makes quantitative analysis of publications based on mathematical and statistical methods. It can scientifically make use of data extracted from articles to analyze and visualize the research hotspots and research frontiers in the identified research domains, which may greatly promote the development of the research field.

## Materials and Methods

The Science Citation Index Expanded (SCI-Expanded) of the Clarivate Analytics Web of Science Core Collection (WoSCC) was the most commonly used scientific information source for bibliometric analysis (13). We had carefully formulated the retrieval plan and selected the last day of 2021 for information screening and extraction, so as to ensure the integrity of the latest research information in 2021 to the greatest extent and avoid the information bias caused by the daily update of the database. The specific search formula was as follows: TS = (“instable patella*” OR “patella* instability” OR “instability of patella*” OR “patella* displacement” OR “displacement of patella*” OR “dislocation of patella*” OR “patella* dislocation” OR “high-riding patella*” OR “patella* alta” OR “lateralized patella*” OR “patella* tilt” OR “subluxation of patella*” OR “patella* subluxation”). The search timespan was set as 2001–2021. We only included two types of literature: original articles and reviews, which had standard references and had been strictly reviewed by experts in the same field. Finally, 2,155 records were finally retrieved. Then we exported records in “plain text” format with “complete records and reference”.

## Data Analysis and Descriptive Analysis

In total, we mainly used 5 scientometric software and Microsoft Excel program to perform bibliometric analysis (Figure 1) (14).

R-bibliometrix software package based on R-Studio (version3.0.3, http://www.bibliometrix.org) (15), VOSviewer software (version1.6.16, https://www.vosviewer.com/download) (16), CiteSpace software (version5.7R5W, https://citespace.podia.com/courses/download) (17), Hiscite software (https://histcite.updatestar.com/) (18) and Pajek software (http://vlado.fmf.unilj.si/pub/networks/pajek/) (19) were employed by the researchers to perform bibliometric analysis.

The VOSviewer software developed by Professor van Eck and Waltman and the CiteSpace software developed by Professor Chen are highly reliable and practical bibliometric software mainly used to visualize and analyze the knowledge structure and the evolution trend of scientific literature in a certain field (20). Besides, Citespace and VOSviewer can extract sub-clusters from the overall structure of literature network through clustering analysis to identify research subdomains, namely research hotspots (21, 22). Nodes in the figures represent countries, institutions, authors, journals, citations or keywords, and links between nodes represent collaboration, co-occurrence or co-citation relationships. We set the nodes in the figures generated as the rainbow ring diagram pattern. The center color of each rainbow node represents the year the study published, and the outer colors represents the years the literature highly cited. The flow of knowledge can be seen from the change of colors in nodes, links and clusters. The warmer the color, the later the year.

In addition, we employed the R-bibliometrix, VOSviewer and CiteSpace software for overlapping analysis to identify the research frontier, which can to a great extent predict the research directions that may produce significant breakthroughs in the next few years. Hiscite and Pajek software are integrated to extract main research trajectories from the huge literature citation network (19, 23). Through in-depth review of the result, researchers can quickly grasp the knowledge base and development trend in the field, so as to improve their own research results and adjust their own research strategies.

## Results and Discussion

### Publication Trends

**Figure 2:**
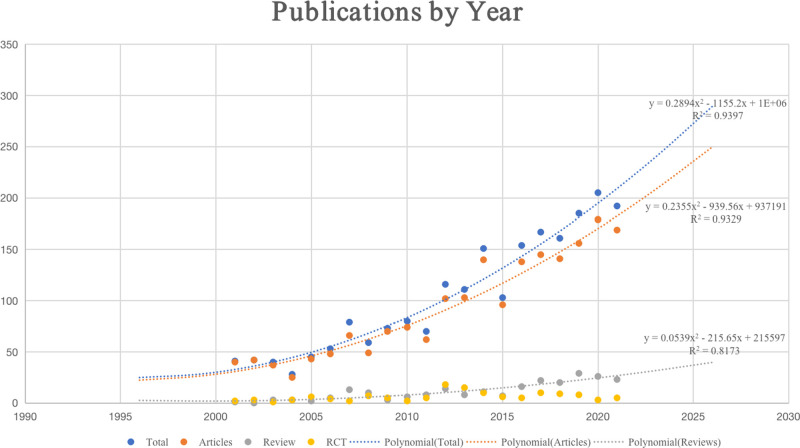
Trends in publications from WOS (2001–2021) by year in the field of patellar instability and the corresponding polynomial fitted curves.

Accessibility to Web of Science, one of the largest citation databases in the world, was obtained through Peking University library. Totally, 2,155 documents related to patellar instability were retrieved. As is demonstrated in Figure 2, the research output before 2001 was relatively low. Despite the appearance of the volatility to decrease at some special time points, we can find a gradually increasing trend year by year after 2001 and the output entered an outbreak growth stage after 2015. Through polynomial fitting analysis between the publication year and the number of publications, we found that there existed a significant correlation (the coefficients of determination (*R*^2^) were 0.9397, 0.9329, and 0.8173 for total documents, articles, and reviews, respectively). According to polynomial fitting analysis, we predicted that the number of papers published in 2025 will reach approximately 270, including about 235 original papers and 35 reviews. In general, the vigorous development of orthopedics and sports medicine makes the research more and more in-depth. However, it can be found that although the number of published publications increases year by year, high-quality RCT researches are still relatively lacking.

## Active Countries/Regions, Institutes and Researchers

**Figure 3:**
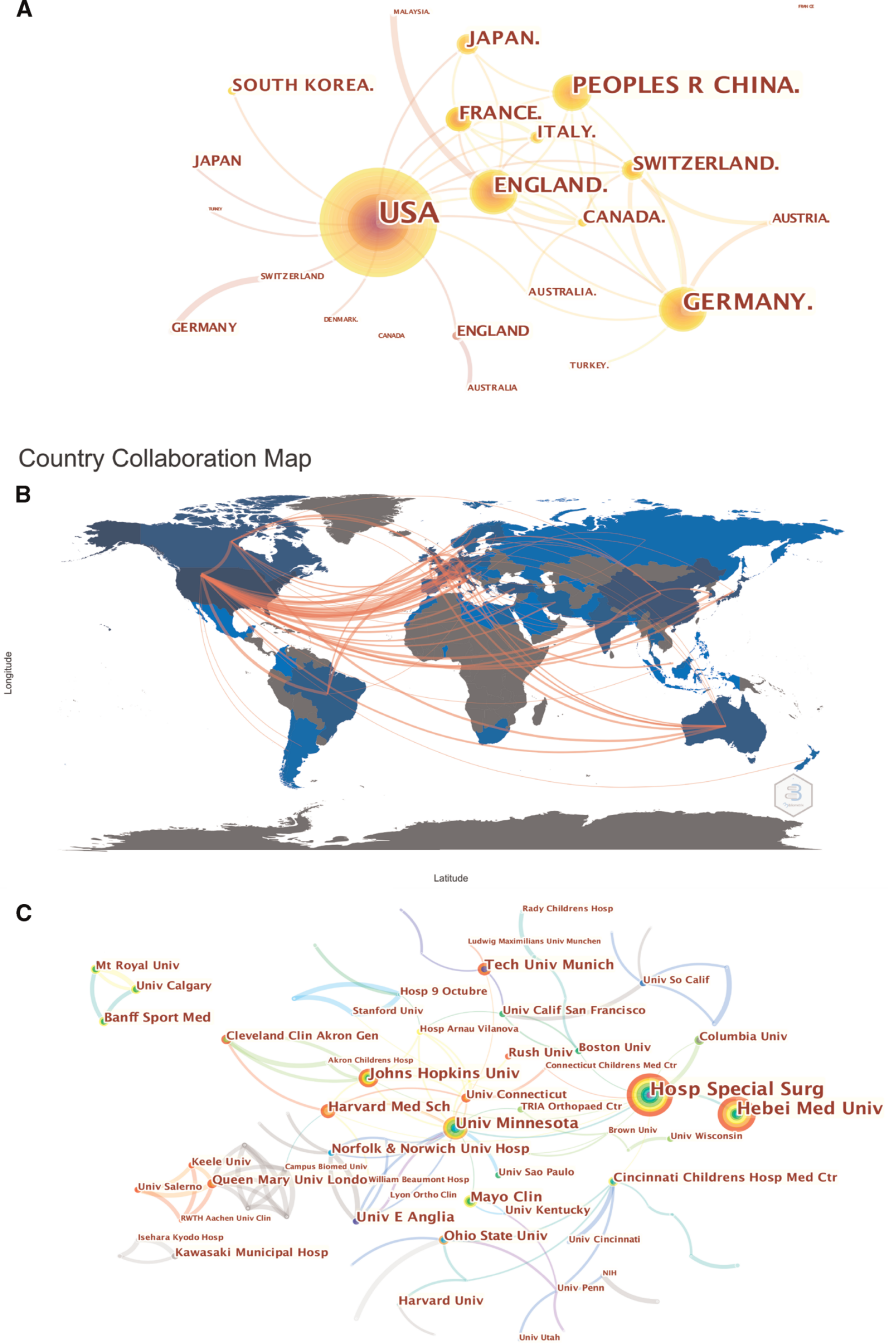
(**A**) The country collaboration network generated by the Citespace software. (**B**) The country collaboration plotted on the world map. (**C**) The collaboration network of institutions generated by the Citespace software.

As is shown in Table 1, the most productive countries and institutions are mainly located in North America, Europe and East Asia. The top 5 influential countries are the United States, Germany, the United Kingdom, Japan and France. In this field, the United States has a pivotal impact, yielding a total of 17,931 citations, far exceeding that of other countries. Figures 3A,B display the international cooperation among different countries worldwide. The Citespace setting parameters of Figure 3A were as follows: # Years Per Slice = 1, Top *N*% = 25, pruning algorithm was adopted. The thickness of the lines between the two countries indicates the cooperation strength. It can be found that extensive cooperative relations have been established among countries in North America, Western Europe and East Asia, but the cooperation among developing countries is still weak and needs to be further strengthened. In addition, it’s necessary for us to note that the average Chinese article citation is not high, whereas the average article citation of Finland is outstanding, suggesting that there is not only a need to improve the quantity but also to seek breakthroughs in the international influence of publications from emerging countries.

**Table 1:** The top 15 influential countries and prolific institutions.

Rank	Country	Total Citation	Average Article Citations	Number of Documents	Affiliations	Articles
1	USA	17,931	25.54	795	Hosp Special Surg	86
2	Germany	4,930	28.66	212	Hebei Med Univ	83
3	UK	4,336	27.44	185	Univ Minnesota	48
4	Japan	2,388	20.07	128	Johns Hopkins Univ	46
5	France	1,902	23.48	109	Tech Univ Munich	37
6	China	1,867	8.45	205	Stanford Univ	34
7	Switzerland	1,784	25.13	113	Univ Zurich	34
8	Finland	1,229	45.52	32	Mayo Clin	33
9	Canada	1,025	19.71	79	Ohio State Univ	31
10	Netherlands	969	22.53	51	Univ Sao Paulo	31
11	Denmark	860	45.26	20	Norfolk and Norwich Univ Hosp	30
12	Australia	830	23.06	62	Med Univ Innsbruck	29
13	Korea	804	12.37	72	Univ E Anglia	29
14	Italy	724	13.92	78	Univ London Imperial Coll Sci Technol and Med	28
15	Austria	557	17.41	49	Univ ULM	28
16	Brazil	552	17.81	41	Harvard Med SCH	27
17	Spain	491	21.35	29	Boston Univ	25
18	Sweden	435	39.55	16	Cincinnati Childrens Hosp Med CTR	25
19	Turkey	415	7.69	61	Harvard Univ	25
20	Belgium	388	24.25	24	Rush Univ	24

Most of these prolific institutions are world-renowned research institutions, with prominent positions in the history of orthopedics and sports medicine research. We can see from the Figure 3C that extensive cooperation between institutions has been established. The Citespace setting parameters of Figure 3C were as follows: # Years Per Slice = 1, Top *N*% = 5, pruning algorithm was adopted. For example, Professor Wang Fei from Hebei Medical University has formed a stable cooperative relationship with the Hospital for Special Surgery. In addition, from the color and size of the nodes, it can be seen that the top 4 prolific institutions are Special Surgery Hospital, Hebei Medical University, University of Minnesota and Harvard Medical School. Focusing on the research of these high-yield institutions and the collaboration can help researchers understand the most significant scientific advances worldwide.

**Figure 4:**
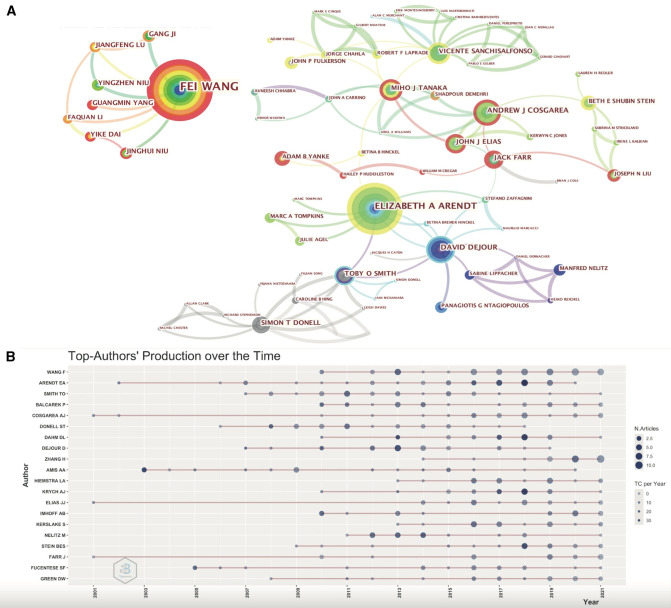
(**A**) The collaboration network of researchers generated by the Citespace software. (**B**) The top twenty prolific researchers in the field and their publications over time. The larger the node, the more articles published. The deeper the color, the more citations. The color represents the number of the publications, and the color represents the citations per year.

Additionally, a total of 318 publications from the top 15 influential authors account for 14.76% of all publications in this field. The author with the highest total citations was Andrew A Amis with 1,586 citations, followed by Philip B Schöttle with 1,265 citations, Elizabeth A Arendt with 1,251 citations and David H Dejour with 1,120 citations. Tables 2, 3 shows the details of the top 15 active researchers, including their H-index (24, 25), total citations, and the relevant information of their representative articles. Collaborations among these authors and their productions over time were shown in Figures 4A,B, respectively. The Citespace setting parameters of Figure 4A were as follows: # Years Per Slice = 1, Top *N*% = 2, pruning algorithm was adopted. It can be found that there are four research clusters, which are radiated by one or two core authors, such as Fei Wang, Vicente Sanchisalfonso, Andrew J Cosgarea, Jack Farr, Elizabeth A Arendt and David Dejour. Generally speaking, the research cluster led by Fei Wang, Andrew J Cosgarea and Jack Farr has been comparatively active in recent years, while the research cluster led by Elizabeth a Arendt and David dejour had greater influence a few years ago.

**Table 2:** The top 15 influential authors.

Rank	Author	H-index	Total citation of the Authors	Number of publications by the authors
1	Amis AA	17	1,586	19
2	Schottle PB	15	1,265	15
3	Arendt EA	20	1,251	35
4	Dejour D	15	1,120	20
5	Powers CM	14	1,096	15
6	Nomura E	12	871	15
7	Balcarek P	12	820	23
8	Dahm DL	13	809	19
9	Donell ST	15	774	21
10	Fucentese SF	12	758	19
11	Smith TO	16	740	25
12	Nelitz M	12	738	17
13	Krych AJ	12	649	18
14	Stuart MJ	13	641	17
15	Wang F	13	530	40

**Table 3:** The most influential articles by the top 15 authors.

Rank	Author	The Most Cited Articles by Each Author	The Journals in which the Top Articles Were Published	Publication Year	Total Citation of the Article
1	Amis AA	Anatomy and biomechanics of the medial patellofemoral ligament	Knee	2003	424
2	Schottle PB	Radiographic landmarks for femoral tunnel placement in medial patellofemoral ligament reconstruction	American Journal of Sports Medicine	2007	333
3	Arendt EA	Current concepts of lateral patella dislocation	Clinics in Sports Medicine	2002	221
4	Dejour D	Osteotomies in patello-femoral instabilities	Sports Medicine and Arthroscopy Review	2007	274
5	Powers CM	Patellofemoral kinematics during weight-bearing and non-weight-bearing knee extension in persons with lateral subluxation of the patella: a preliminary study	journal of Orthopaedic & Sports Physical Therapy	2003	199
6	NOmura E	Long-term follow-up and knee osteoarthritis change after medial patellofemora ligament reconstruction for recurrent patellar dislocation	American Journal of Sports Medicine	2007	119
7	Balcarek P	Anatomy of lateral patellar instability trochlear dysplasia and tibial tubercle-trochlear groove distance is more pronounced in women who dislocate the patella	American Journal of Sports Medicine	2010	120
8	Dahm DL	Predictors of recurrent instability after acute patellofemoral dislocation in pediatric and adolescent patients	American Journal of Sports Medicine	2013	160
9	Donell ST	Acute patellar dislocation in children and adolescents: a randomized clinical trial	Journal of Bone and Joint Surgery-American Volume	2008	240
10	FucentesE SF	Clinical and radiological outcome of medial patellofemoral ligament reconstruction with a semitendinosus autograft for patella instability	Knee Surgery Sports Traumatology Arthroscopy	2005	221
11	Smith TO	Operative versus non-operative management of patellar dislocation. a meta-analysis	Knee Surgery Sports Traumatology Arthroscopy	2011	74
12	Nelitz M	Observer agreement on the dejour trochlear dysplasia classification a comparison of true lateral radiographs and axial magnetic resonance images	American Journal of Sports Medicine	2012	120
13	Krych AJ	CT and MRI measurements of tibial tubercle-trochlear groove distances are not equivalent in patients with patellar instability	American Journal of Sports Medicine	2013	123
14	Stuart MJ	CT and MRI measurements of tibial tubercle-trochlear groove distances are not equivalent in patients with patellar instability	American Journal of Sports Medicine	2013	123
15	Wang F	Functional bundles of the medial patellofemoral ligament	Knee Surgery Sports Traumatology Arthroscopy	2010	90

It is worth noting that one of the most influential authors–Andrew A Amis is a professor from the Department of Mechanical Engineering in Imperial College London, focusing on biomechanics for orthopedics (26). The comprehensive research of medicine and engineering has gradually become the hotspots, which can make a major breakthrough that is difficult to be achieved through traditional clinical researches (27).

With David H Dejour as the representative, Lyon School stands out as one of the notable landmarks in the progress of the research of patellar instability. Through the research and summary of several generations of scholars, Lyon School had established the theory that the abnormality of bone structure is the basic cause of patellar instability, and they formulated quantitative standards for radiographic detection and surgical indications for osseous risk factors (6, 28). In the last 20 years, soft-tissue surgeries such as medial patellofemoral ligament reconstruction have gradually become the worldwide research hotspot (29), which led to the formation of the America School with soft-tissue reconstruction as their core concept. And it can be seen from Figure 4 that Chinese scholars played an increasingly significant role in this field. For example, Professor Fei Wang made outstanding contributions in both clinical and preclinical medicine research on patellar instability.

### Journals

**Figure 5:**
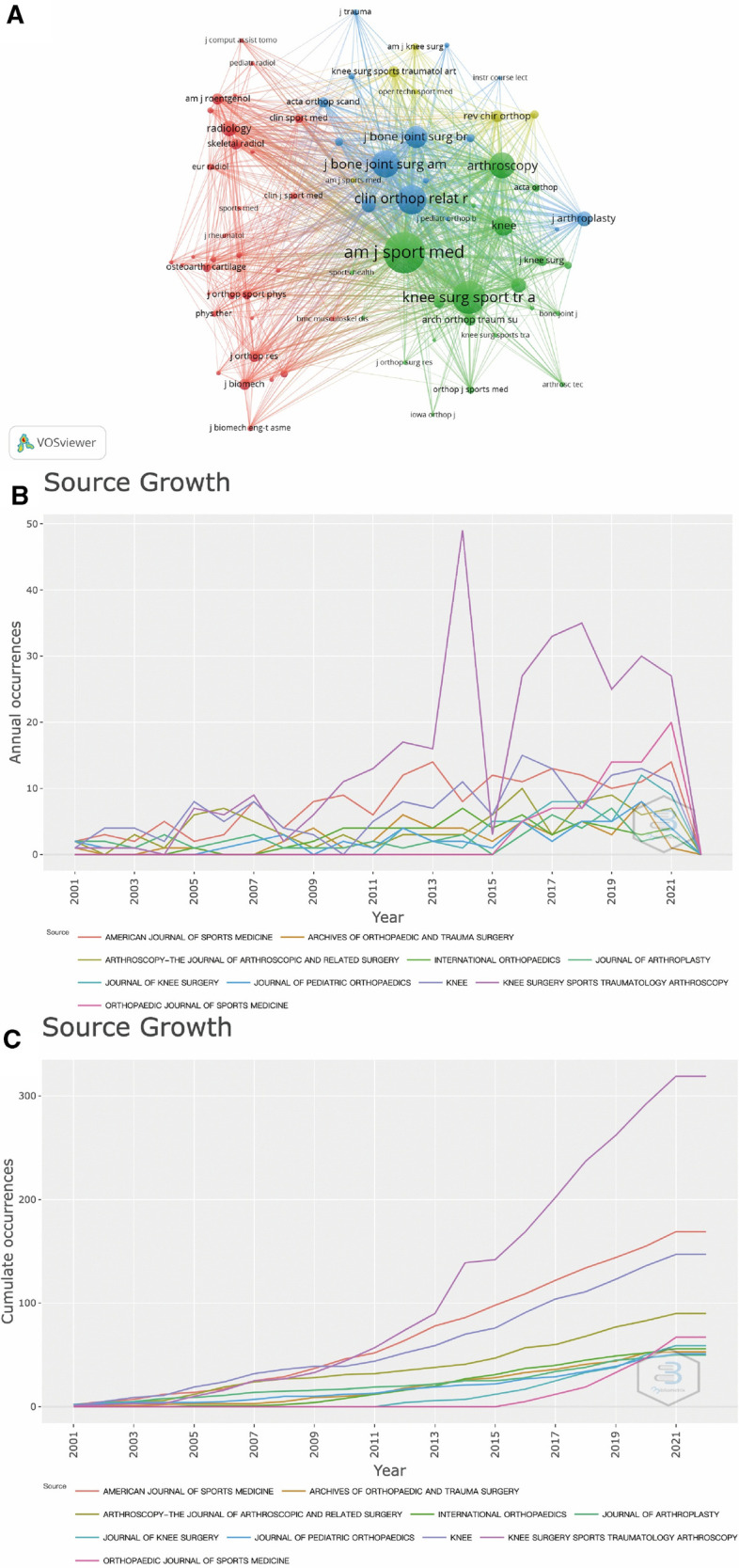
(**A**) Cluster visualization of the journal co-citation analysis generated by the VOSviewer software. Each node represents a journal, and the size of each circle is determined by the co-citations of the journal. (**B**) Annual publication trend of the prolific journals (**C**) Cumulative publication trend of the top prolific journals.

Scientific publications are important carriers of knowledge in a specific field. The statistical analysis of the distribution of the journal source can help researchers choose the most appropriate journals to publish their research findings, and also help the most relevant journal sources to obtain more abundant manuscript submission. The top 20 journals were presented in Table 4. Visualization of the journal co-citation analysis was shown in Figure 5 (30). The journal with the largest H-index and the largest total citations is the American Journal of Sports Medicine (H-index = 58, total citations = 14,370) while the journal with the largest number of publications is Knee Surgery Sports Traumatology Arthroscopy (Number of Publications, NP = 311).

**Table 4:** The most 20 prolific journals in the field.

Rank	Source	H-index	Total Citation	Number of Publications	Quartile in category (2020)
1	Knee Surgery Sports Traumatology Arthroscopy	44	7,314	311	Q1
2	American Journal of Sports Medicine	55	8,983	163	Q1
3	Knee	25	2,657	134	Q3
4	Arthroscopy-the Journal of Arthroscopic And Related Surgery	34	3,499	88	Q1
5	Archives of Orthopaedic and Trauma Surgery	16	979	56	Q2
6	International Orthopaedics	20	1,418	55	Q2
7	Journal of Knee Surgery	13	607	55	Q2
8	Journal of Pediatric Orthopaedics	16	711	49	Q3
9	Orthopaedic Journal of Sports Medicine	10	326	49	Q2/Q3
10	Journal of Arthroplasty	16	840	47	Q1
11	Clinical Orthopaedics and Related Research	24	2,221	43	Q1
12	Sports Medicine and Arthroscopy Review	16	966	39	Q3
13	Skeletal Radiology	14	799	32	Q3
14	Journal of Bone and Joint Surgery-American Volume	17	1,628	29	Q1
15	Orthopaedics & Traumatology-Surgery & Research	11	410	29	Q3
16	Journal of Bone and Joint Surgery-British Volume	17	1,405	22	Q1
17	Journal of Orthopaedic Research	14	867	22	Q1
18	Bone & Joint Journal	11	257	16	Q1
19	Clinics in Sports Medicine	12	537	14	Q3
20	Journal of Biomechanics	10	366	13	Q3

In addition to these two journals, the top ten influential journals include: Knee (NP = 134), Arthroscopy-the Journal of Arthroscopic and Related Surgery (NP = 88), Archives of Orthopaedic and Trauma Surgery (NP = 56), International Orthopaedics (NP = 55), Journal of Knee Surgery (NP = 55), Journal of Pediatric Orthopaedics (NP = 49), Orthopaedic Journal of Sports Medicine (NP = 49), Journal of Arthroplasty (NP = 47). Scientific achievements with major breakthroughs in the future are likely to appear in these journals.

**Figure 6:**
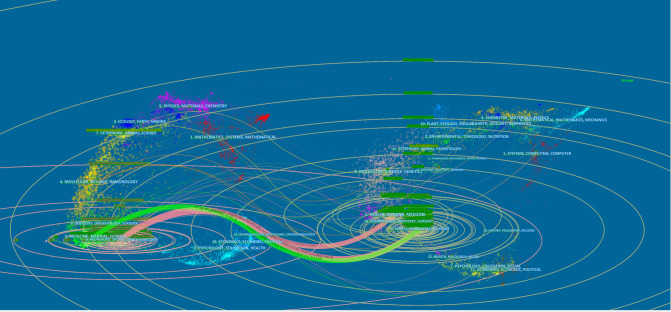
The dual-map overlay of journals contributed to publications on patellar instability from 2001 to 2021.

As is shown in Figure 6, each point represents a journal, and the most influential journals are represented by ellipses instead - the center of the ellipses represent the subject field, the horizontal axis of the left ellipses represents the number of authors, and the vertical axis represents the number of publications. Meanwhile, the horizontal axis of the right ellipses represents the number of cited authors, and the vertical axis represents the number of times the journal has been cited. Besides, the journals are grouped into clusters by adopting the Blondel algorithm to identify the major research disciplines (31), because the citing papers are regarded as the frontiers of knowledge and the cited papers are considered as the basis of knowledge.

In addition, the colored paths between the clusters of journals in the dual-map overlay indicate the citation relationships between the citing journals and the cited journals, which demonstrate the citation trajectory and knowledge flow of knowledge (32). The colored paths indicated that studies published in Medicine/Medical/Clinical/Surgery journals usually cite the studies published in Sports/Rehabilitation/Surgery, Health/Nursing/Medicine, Molecular/Biology/Genetics and Forensic/Anatomy/Medicine. More information about the representative citing and citied journals in each cluster can be detected in Figure 6. For instance, the most representative journals in the Health/Nursing/ Medicine cluster are the Clinical Orthopaedics and Related Research, the Journal of Bone and Joint Surgery, the Arthroscopy, the Knee and the International Orthopeadics.

**Figure 8:**
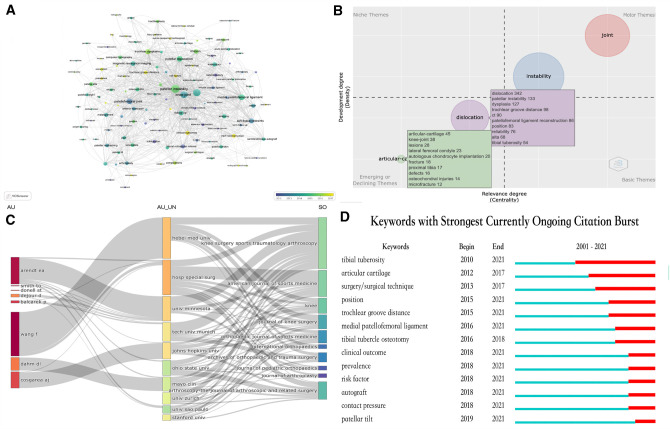
(**A**) Mapping of keywords based on keywords co-occurrence analysis. (**B**) Thematic map of Keywords generated by the Biblioshiny app. (**C**) The three-field plot showing the knowledge flow. (**D**) Keywords with strongest currently ongoing citation burst.

In addition, the Figure 8C shows the three-field plot generated by R-Bibliometrix software package, which can intuitively show the flow of knowledge - including the cooperative relationship between the most productive authors and the most productive institutions, as well as their most preferred journal sources to publish their scientific findings.

### References

Reference is one of the most significant aspects of bibliometrics. We mainly analyzed the co-citation information of the articles from two aspects (33).

#### Intellectual base and main research trajectories of the patellar instability research field

**Figure 7:**
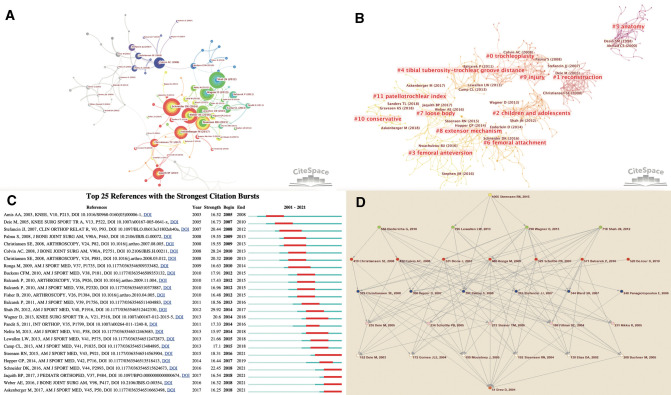
(**A**) Intellectual base of research on patellar instability. Note: It can be seen that the influential literature has gradually increased in recent years, and more links have been generated. (**B**) The top 25 references with the strongest citation bursts. (**C**) Cluster visualization of the co-citation network of references via Citespace, together with the details and the representative references of the generated clusters. Note: This figure is arranged in chronological order from left to right. (**D**) The research main path during 2001–2021. Note: The research main path analysis is performed based on the algorithm of the Pajek software.

Frequently cited literatures usually have great influence in the relevant research fields. As is shown in Figure 7A, a co-citation reference network with 105 nodes was fabricated to demonstrate the most significant studies. The selection criteria were set as follows: # Slice Length = 1 year, Top1% per slice, pruning algorithm was adopted. This reference network with a density of 0.0211 contains 21 colors from light gray to bright red representing different years. From the links, that is, the co-citation relationship, we can see the development process of the research field and the correlation between the influential literatures. Moreover, burst detection, an algorithm developed by Kleinberg (Bursty and Hierarchical Structure in Streams), was an effective analytic tool to capture the sharp increase of references or keywords popularity within a specified period (34). This function can serve as an efficient way to identify concepts or topics that were actively discussed during some period of time. Figure 7B lists the top 25 references with the strongest citation bursts in chronological order, and the citation strength is represented by the size of the nodes in Figure 7A. Reading these papers can help researchers grasp the basis of knowledge more intuitively and quickly.

It can be seen that the most influential reference is Shah JN, 2012 (4), which systematically quantifies the postoperative complications of medial patellofemoral ligament reconstruction in patients with patellofemoral instability - the major complications are patellar fracture, postoperative instability, flexion loss and pain. Among the references with citation burst lasted until 2021, the publication with highest strength was Schneider DK, 2016 (35), which provided a wide range of surgical criteria for isolated medial patellofemoral ligament reconstruction through systematic review and meta-analysis.

Tracking the main research trajectories of a small research field may not be a difficult task, because the scholars don’t have to spend a lot of time reviewing a large number of literatures. However, when it is come to study a large research field, it is more difficult and significant for researchers to track the research trajectories. Professor Liu introduced a quantitative method, namely, main path analysis (36), which simplifies large and complex research fields into one or several main trajectories, which are composed of several key nodes and links, as shown in Figure 7D (for details of the main research trajectories, see Supplementary Table S1).

It can be found that the last node is Steensen R, 2015 (37), which closes all the main trajectories. The significant article analyzed the anatomical factors related to recurrent patellar dislocation through a magnetic resonance imaging study, and put forward the future research direction - the accuracy of risk factor model.

The articles on the main trajectories mainly focus on: (1) epidemiological research and recurrence prediction (38–40), risk factors according to radiography (41–43) of patellar instability; (2) indications (44), surgical techniques (45, 46), clinical prognosis (47–55) and biomechanics research (56, 57) of medial patellofemoral ligament reconstruction;(3) indications and prognosis of osseous operation, such as osteotomy and trochleoplasty (58, 59);(4) the indications for conservative treatment——the difference between prognosis of surgery and conservative treatment (60–62) (5) biomechanical research of knee joint——the posterior stabilizer (63) and the stability of Patellar Alta (64). Besides, it can be found that high-quality RCTs and systemic reviews are more likely to have a significant impact on the research field.

#### A Co-Cited Documents-Based Clustering Analysis

A co-cited documents-based clustering analysis can present subfields which represent the main research hotspots (22). Figure 7C presents the clusters of the co-citation network of references: “trochleoplasty (cluster #0),” “reconstruction (cluster #1),” “children and adolescents (cluster#2),” “femoral anteversion (cluster#3),” “tibial tuberosity-trochlear groove distance (cluster#4),” “anatomy (cluster#5),” “femoral attachment (cluster#6),” “loose body (cluster#7),”“extensor mechanism (cluster#8),” “injury (cluster#9),” “conservative (cluster#10)” and “patellotrochlear index (cluster#11). The cluster setting parameters were as follows: # Years Per Slice = 1, Top *N*% = 5, pruning algorithm was adopted. The Modularity Q score was 0.7123, >0.5 and the Weighted Mean silhouette score was 0.8911 > 0.5, indicating the network was reasonably divided into loosely coupled clusters and the homogeneity within the clusters was credible.

From the change of colors, we can divide the research field into four development stages: Stage I (before 2000): the research mainly focuses on the study of anatomy, which is also the time period during which the French Lyon School made outstanding contributions to the foundation of the research field (65, 66); Stage II (2000–2010): the research mainly focused on trochleoplasty (osseous surgery), medial patellofemoral ligament reconstruction (soft tissue repair) (1, 67, 68); Stage III (2011–2013): the study mainly focused on the indications and complications of surgery in children and adolescents and the application value of TT-TG distance in diagnosis and treatment (69–71); Stage IV (2013–present): the research has gradually deepened, and the research focus has gradually shifted from the femoral attachment and knee extension mechanism(2013–2015) to the treatment of loose body after injury and femoral anteversion (2016–2017), and finally patellotrochlear index and conservative treatment (2018–present) (2, 37, 72).

### Keywords–Co-Occurrence and Research Frontier Analysis

In addition to the reference co-citation analysis, keyword co-occurrence analysis can help us identify the main topics and core contents (73). Therefore, it has become another important research strategy of bibliometrics. Co-occurrence analysis mainly determines the relationship between keywords according to the number of keywords appearing together in a literature. We conducted cleaning and calculation of the data by using VOSviewer software (74), and after setting a selection threshold of 18 for the number of keyword occurrences, we identified 154 relevant keywords. Figure 8A shows the overlaying visualization of core keywords in the patellar instability research. The size of nodes reflects the occurrence times of keywords, and the distance between two nodes is directly proportional to the correlation strength between keywords. Besides, all these keywords are also marked with different colors. Relatively early keywords are colored in blue, while recent keywords are colored in yellow.

As is shown in Figure 8B, the thematic map generated by R-Bibliometrix software package is displayed in the form of a two-dimensional matrix. The two dimensions of the matrix - centrality and density are represented by the X-axis and Y-axis respectively. The X-axis represents the centrality, that is, the significance of the subject, and the Y-axis represents the density, that is, the centrality of the subject. Accordingly, the upper right quadrant (i.e., quadrant 1) pertains to motor themes that are both important and well-developed, the upper left quadrant (i.e., quadrant 2) is associated with highly developed and isolated themes, the lower left quadrant (i.e., quadrant 3) refers to emerging or declining themes, and the lower right quadrant (i.e., quadrant 4) contains transversal and basic themes. It can be found that there exist two keyword bubbles in the quadrant of emerging or declining themes.

Distinct software based on different algorithms will generate results laying particular emphasis on different aspects, the thematic map generated by the R-Bibliometrix software package, the keyword burst analysis of CiteSpace and the overlapping visualization of VOSviewer are comprehensively utilized (10), so as to accurately identify the frontier in the research field of patellar instability (24). As is shown in Figure 8, after overlapping analysis, there are 7 keywords that are identified as potential research frontiers—MPFL (medial patellofemoral ligament) construction, clinical outcome, risk factors, prevalence, articular cartilage, tibial tuberosity and tibial tubercle-trochlear groove (TT-TG) distance. Focusing on making breakthroughs in these research directions will likely yield significant research findings that will greatly give impetus to the field

#### MPFL (Medial Patellofemoral Ligament) Construction, Tibial Tuberosity and Tibial Tubercle-Trochlear Groove (TT-TG) Distance

We can find that soft tissue reconstruction (medial patellofemoral ligament construction) and osseous surgery (tibia tubercle anteriomedialis transfer) are still the main research frontiers. Medial patellofemoral ligament construction is one of the most concerned surgical procedures. The gradual deepening of people's understanding of the anatomy and biomechanics of the complex medial patellar retinaculum (75): the proximal medial patellar restraints (MPFL and medial quadriceps tendon-femoral ligament) and the distal medial patellar restraints (medial patellatibial ligament and medial patella meniscal ligament) provides new insight for the medial patellofemoral ligament construction (76). However, the complexity of patellofemoral joint movement and individual differences of patients lead to great disputes among scholars on the selection of attachment point, the tension of reconstructed ligament and MPFL anisometry (77), which are critical factors that influence the overall outcome after MPFL reconstruction.

#### Clinical Outcome, Risk Factors and Prevalence

The establishment of multivariate prediction model of patellar instability is difficult but full of potential because of the complex and diverse risk factors of the disease. A variety of variables to consider include: general risk factors such as age, gender, family history, history of congenital dislocation of the hip (78); morphological risk factors included trochlear dysplasia, patellar Alta, a laterally placed tubercle and patellar tilt (79); In addition, the results of physical examination and imaging risk factors need to be included. These variables are important perioperative indicators that do not involve surgical technology. The establishment of multivariable model and the application of radiomics can provide long-term and reliable medical management for patients (80), so as to prevents morbidity in high-risk patients and improve the prognosis of patients to a certain extent. However, due to the lack of the number of clinical cases and long-term and reliable clinical research results, the establishment of the model is still difficult, but some breakthroughs have been made. For example, Duerr, Robert A established an algorithm model to manage recurrent patellar dislocation (81).

In the choice between conservative management and surgical treatment, including MPFL reconstruction, trochleoplasty, tibial tubercle transfer and femoral rotational osteotomy, we need to comprehensively consider the complications of the treatments, the probability of recurrence and the performance of patients returning to exercise. Therefore, the clinical outcomes of treatment have been the research focus of researchers for a long time, and therefore can produce more influential articles in the future.

#### Articular Cartilage

Patellofemoral joint can cause cartilage injury in the early stage, leading to the development of osteoarthritis. Most surgical operations only focus on the treatment of patellar instability, do not treat the damaged cartilage, and there is a lack of corresponding high-level clinical research (82). The treatment of cartilage is a very dynamic research frontier. In addition to conventional arthroscopic chondral debridement, the rapid development of technologies and biomaterials makes this field full of possibilities, including autologous matrix-induced chondrogenesis (AMIC) combining microfracture with collagen I / III matrix (83), autologous osteochondral transplantation or inlay, and the introduction of matrix-assisted autologous chondrocyte transplantation (MACT) procedure in the first-generation of autologous chondrocyte implantation (ACI) (84), and cartilage regeneration technology of osteochondral scaffolds (85). It can be expected that in the future, with the in-depth understanding of the etiology and biomechanics of cartilage degenerative diseases, more advanced and effective treatments will appear, so as to obtain great influence.

## Limitations

There are still some limitations in our current research.

Firstly, there are certain limitations in our study. Firstly, in our study, we only searched the Web of Science Core Collection (WoSCC) and did not incorporate other databases, such as PubMed, Scopus or Embase. However, it may be unscientific to merge and analyze the data from multiple databases, because different databases have different measurement of citation frequency counting and classification of publications (9, 86).

Secondly, only English publications were included, which may lead to the omission of a portion of high-quality articles published in other languages.

Finally, there existed two potential disadvantages: (1) there was no manual cleaning of the sample data before formal analysis; (2) because the analyses were completed by software, there might be some errors or biases in our results. For example, the name of the journals may be changed over a long period of time. In addition, two authors with the same name may be repeatedly accumulated.

## Conclusions

This is the first comprehensive bibliometric analysis of patellar instability. Our results show that patellar instability has gradually attracted the attention of scholars, which can be seen from the increasing number of articles and citations year by year. So far, the United States has been in a leading position in this field. Hospital for special surgery and Andrew A Amis are the most prolific institutions and the most influential authors respectively. The American Journal of Sports Medicine and Knee Surgery Sports Traumatology Arthroscopy are the most influential and productive journals in the study of patellar instability, with the most citations and publications respectively. According to the analysis of references, we identified 11 research hotspots of patellar instability in chronological order, and identified the main research paths in this field. In addition, the comprehensive analysis of keywords identified mpfl (medial patellofemoral ligament) construction, clinical outcome, risk factors, prevalence, articular cartilage, tibial tuberosity and tibial tubercle-trochlear groove (TT-TG) distance as significant research directions in the future, which deserves the attention of researchers. In short, scholars, especially researchers newly entering this industry, can benefit from our research, which can enable them to clearly and quickly understand the global hotspots, trends and knowledge structure of this field, so that they can be inspired to a certain extent.

## Data Availability

The original contributions presented in the study are included in the article/Supplementary Material, further inquiries can be directed to the corresponding author/s.
